# Can Mesopic Virtual Reality Detect Changes Prior to Traditional SITA Humphrey Visual Fields in Neuro-Ophthalmology Patients?

**DOI:** 10.51894/001c.144116

**Published:** 2025-09-30

**Authors:** Emma Proctor

**Affiliations:** 1 College of Osteopathic Medicine Michigan State University

## 36

### INTRODUCTION

Early detection of visual field defects in Neuro-Ophthalmologic disorders like pituitary adenoma, optic neuritis and idiopathic intracranial hypertension can lead to earlier therapeutic intervention. Visual field defects are traditionally detected using Humphrey Visual Fields (HVF), which employ a photopic (cone-dominated) luminance. We compared this gold standard against a mesopic (cone and rod luminance) method using a Virtual Reality (VR) headset.

There is very little data available comparing longitudinal use of VR to traditional HVF. We aimed to determine if VR fields were comparable or additive to serially performed HVFs. We found that VR fields are as reliable as HVF, and even have the potential for earlier detection.

### METHODS

The HVF uses the photopic Swedish Interactive Threshold Algorithm (SITA). The patient sits with their entire head in a rounded globe-like apparatus while a computer-assisted paradigm administers the test. The commercially available “VF3” VR device uses the mesopic Block’s Optimal Luminance Thresholding (BOLT), in which the patient uses googles only to completely cover both eyes.

We evaluated seven controls (14 eyes) and seven patients (13 eyes) longitudinally with both HVF and VR on the same day. All tested between 1-20 weeks (average eight weeks) after their initial visit to evaluate the reproducibility of the VF3. Reliability indices, mean deviation (MD), and correlation of total deviation p-values of 0.05 (indicating a visual field defect in a given location) were recorded. A questionnaire to determine preference was administered.

### RESULTS

All 14 normal eyes changed similarly for MD and total deviation p-value abnormalities on VR and HVF between visits. Eight of 13 patient eyes changed similarly between VR and HVF tests based on p-values. Of the 5 eyes that didn’t change similarly, VR showed potential earlier change than HVF did. Changes in MD were similar in 10 of 13 patient eyes. HVF was preferred in 4/7 normals. VR was preferred in 6/7 patients.

### CONCLUSION

The VF3 device has the potential to detect visual deficits earlier than HVFs, perhaps based on use of a mesopic background. Further prospective studies are needed to determine the ultimate longitudinal testing value of VR.

